# *KRAS* and *BRAF* mutations in circulating tumour DNA from locally advanced rectal cancer

**DOI:** 10.1038/s41598-018-19212-5

**Published:** 2018-01-23

**Authors:** Francesco Sclafani, Ian Chau, David Cunningham, Jens C. Hahne, George Vlachogiannis, Zakaria Eltahir, Andrea Lampis, Chiara Braconi, Eleftheria Kalaitzaki, David Gonzalez De Castro, Andrew Wotherspoon, Jaume Capdevila, Bengt Glimelius, Noelia Tarazona, Ruwaida Begum, Hazel Lote, Sanna Hulkki Wilson, Giulia Mentrasti, Gina Brown, Diana Tait, Jacqueline Oates, Nicola Valeri

**Affiliations:** 1 The Royal Marsden NHS Foundation Trust, London and Surrey, United Kingdom; 2 The Institute of Cancer Research, London and Surrey, United Kingdom; 3 Vall d’Hebron University Hospital, Universitat Autònoma de Barcelona, Barcelona, Spain; 4 University of Uppsala, Uppsala, Sweden; 5 Biomedical Research Institute INCLIVA, University of Valencia, Valencia, Spain

## Abstract

There are limited data on circulating, cell-free, tumour (ct)DNA analysis in locally advanced rectal cancer (LARC). Digital droplet (dd)PCR was used to investigate *KRAS/BRAF* mutations in ctDNA from baseline blood samples of 97 LARC patients who were treated with CAPOX followed by chemoradiotherapy, surgery and adjuvant CAPOX ± cetuximab in a randomised phase II trial. *KRAS* mutation in G12D, G12V or G13D was detected in the ctDNA of 43% and 35% of patients with tumours that were mutant and wild-type for these hotspot mutations, respectively, according to standard PCR-based analyses on tissue. The detection rate in the ctDNA of 10 patients with less common mutations was 50%. In 26 cases ctDNA analysis revealed *KRAS* mutations that were not previously found in tissue. Twenty-two of these (84.6%) were detected following repeat tissue testing by ddPCR. Overall, the ctDNA detection rate in the *KRAS* mutant population was 66%. Detection of *KRAS* mutation in ctDNA failed to predict prognosis or refine patient selection for cetuximab. While this study confirms the feasibility of ctDNA analysis in LARC and the high sensitivity of ddPCR, larger series are needed to better address the role of ctDNA as a prognostic or predictive tool in this setting.

## Introduction

Screening for tumour genetic alterations is a standard procedure in modern oncology. Analysis of biopsy and/or resection specimens is routinely performed in many cancers in an attempt to identify molecular abnormalities that can provide useful diagnostic, prognostic or predictive information and assist clinicians in the decision-making process.

Nevertheless, it has been increasingly recognised that tissue-based genetic tests are limited by some inherent characteristics of cancer such as intra-tumour heterogeneity and clonal evolution^1^. Tumour lesions are composed of clones of cancer cells that may differ in terms of genetic make-up and aggressive potential. The relative contribution of each clone to the overall tumour phenotype and disease burden at a given time is also largely affected by a number of factors including exposure to treatments that exert a selective pressure on cancer cells and ultimately drive tumour evolution^2^. Therefore, genetic analyses on pre-existing archived tissue and/or random sampling of small amount of tumour may result in a suboptimal portrayal of the tumour molecular profile and be of limited value in routine practice.

Over the last few years, detection and analysis of circulating, cell-free, tumour DNA (ctDNA) in the blood has emerged as an alternative analytic method with the potential to overcome the above limitations and provide a real-time, exhaustive characterisation of cancer genome^3^. Although the exact mechanisms whereby cancer cells shed DNA into the bloodstream have not been fully elucidated, it is now clear that a simple blood sample (i.e., liquid biopsy) is a valuable source of genetic material likely to encompass the wide intra- and inter-lesional tumour heterogeneity^4^. Compared with conventional, tissue-based, sampling procedures, blood sampling is quicker, less invasive and by far more convenient for both patients and clinicians/health providers. All these advantages make liquid biopsy also particularly suitable for the dynamic assessment of tumour response to treatment or monitoring of disease status during follow-up^5–7^. The clinical usefulness of ctDNA analysis is confirmed by the recent approval by the Food and Drug Administration of the cobas EGFR Mutation Test v2 as a blood-based diagnostic tool for the detection of epidermal growth factor receptor (*EGFR*) mutations and selection of non-small cell lung cancer patients who are candidates for erlotinib treatment^8^. Also, algorithms have been proposed for non-invasive diagnosis and discrimination of cancer type based on copy number variation in ctDNA^9^.

In colorectal cancer, detecting and quantifying ctDNA by using common somatic mutations (i.e., *APC*, *TP53*, *KRAS*) and/or tumour epigenetic alterations (i.e., *CDKN2A* or *RASSF2A* methylation) has been demonstrated to be feasible and clinically relevant. Studies have reported an association between presence of post-operative ctDNA and risk of tumour recurrence in early-stage colon cancer^10–12^ or presence/levels of ctDNA and overall tumour burden/prognosis in metastatic patients^13,14^. Moreover, tracking *KRAS* mutations and other genetic aberrations accounting for primary or secondary resistance to anti-EGFR monoclonal antibodies is an attractive dynamic method to monitor the emergence/evolution of resistant clones to cetuximab or panitumumab and to potentially allow the implementation of adaptive treatment strategies^4,15–17^.

Nevertheless, limited information is available on the feasibility and clinical potential of ctDNA analysis in non-metastatic rectal cancer. Algorithms for risk stratification have been recently developed for these patients and risk-adapted therapies increasingly investigated in clinical trials and ultimately implemented in routine practice^18–20^. It is possible that, in this setting, the analysis of ctDNA may provide valuable information to combine with standard clinico-pathological and imaging data and lead to a better assessment of individual patient risk and more refined treatment approaches. Only a few studies have been conducted so far in rectal cancer patients who were treated with neoadjuvant chemoradiotherapy and surgery^21–24^. The results of these studies are difficult to interpret and compare especially due to the small numbers and significant heterogeneity with regard to the methods used for the assessment of ctDNA (i.e., total circulating cell-free DNA, DNA integrity index or tumour-specific molecular alterations) and the outcome measures selected for the evaluation of tumour response to treatment (i.e., pathological downstaging or tumour regression grading).

Therefore, we aimed to contribute to the existing data around liquid biopsy in non-metastatic rectal cancer by assessing feasibility, hurdles and potential clinical implications of using *KRAS/BRAF* mutations as markers for ctDNA detection in a relatively large prospective series of locally advanced rectal cancer (LARC) patients who were included in a randomised phase II trial (EXPERT-C)^25^.

## Material and Methods

### Patients

EXPERT-C (Trial registration: ISRCTN Register: 99828560) was an international, multicenter, randomised phase II trial in which patients with high-risk LARC were treated with 12 weeks of neoadjuvant chemotherapy with CAPOX (oxaliplatin 130 mg/m^2^ on day 1 and capecitabine 1700 mg/m^2^/day for 14 days, every 3 weeks) followed by chemoradiotherapy (50.4 Gy in 28 fractions with concomitant continuous capecitabine 1650 mg/m^2^/day over 6 weeks), total mesorectal excision (4 to 6 weeks after completion of chemoradiotherapy) and 12 weeks of adjuvant CAPOX (as above) with or without weekly cetuximab (400 mg/m^2^ loading dose, 250 mg/m^2^ subsequent doses)^25^. Randomisation was in a 1:1 ratio. Written informed consent was obtained from each patient before study entry.

In this study, the definition of high-risk locally advanced tumour was based on high-resolution MRI of the pelvis. Only patients who had at least one of the following high-risk features at baseline were considered eligible: depth of extramural spread ≥5 mm (i.e., so called T3c or T3d stage), T4 stage, T3 tumour at/below levator muscles, tumour within 1 mm of mesorectal fascia, or presence of extramural venous invasion (EMVI). Distant metastases were an exclusion criterion and ruled out with a CT scan of the thorax and abdomen. During treatment, MRI and CT scans were repeated after completion of neoadjuvant chemotherapy and chemoradiotherapy with MRI scans being centrally reviewed by a blinded radiologist. Details regarding patient follow-up have been reported previously^25^.

### Blood-based *KRAS* and *BRAF* mutation analysis

DNA was isolated from 2 ml of plasma collected prior to commencement of neoadjuvant treatment and analysed by digital droplet (dd)PCR (QX200 ddPCR system, Bio-Rad, Berkeley, California) according to the manufacturer’s protocol. All PCR reactions were prepared using the ddPCR Supermix with no dUTTP for probes (Bio-Rad) and performed as duplex PCR using the relevant digital PCR assays for the wild-type and the mutation in question. Droplets were generated using the QX200 droplet generator. The PCR reaction was performed in a C1000 Touch Thermo Cycler (Bio-Rad) using the following protocol: 95 °C for 10 minutes followed by 40 cycles of 94 °C for 30 seconds and 55 °C for 1 minute, then 98 °C for 10 minutes. Droplets were read in the QX200 droplet reader and analysed using the Quantasoft software version 1.6.6.0320 (Bio-Rad).

Due to the limited availability of plasma, only the most common *KRAS* (i.e., G12D, G12V and G13D) and *BRAF* (i.e., V600E) mutations were analysed in all assessable patients. Any additional, patient-specific, *KRAS* mutation which was previously detected in the tumour tissue was also analysed in the DNA extracted from the plasma. The percentage of mutant *KRAS* or *BRAF* alleles (i.e., fractional abundance) was calculated as the ratio of drops positive for the mutant allele to drops positive for the mutant allele plus drops positive for the wild-type allele. The sensitivity cut-off for the ctDNA detection assay was set at the lower limit of 0.02% mutant alleles as previously reported^26^.

### Tissue-based *KRAS*/*NRAS/BRAF* mutation analysis

When this study was conducted, data on tumour *KRAS*, *NRAS* and *BRAF* status for all assessable patients were already available. DNA for these mutational analyses was extracted from formalin-fixed, paraffin-embedded (FFPE) tissue sections from biopsy and/or resection samples (depending on tissue availability/quality) and analysed in a central laboratory using standard PCR-based techniques. All the analyses were performed by investigators who were blinded to the clinical data. *KRAS* (exon 2 and 3) and *BRAF* (V600E) mutations were prospectively analysed with the INFINITI platform (AutoGenomics, Vista, CA, US). *NRAS* (exon 3) mutations were screened for as part of the original translational sub-study using multiplex PCR. Finally, *KRAS* (exon 4) and *NRAS* (exon 2 and 4) mutations were retrospectively analysed bi-directional Sanger sequencing. All mutations detected were confirmed on an independent PCR and sequencing analysis. For the purpose of this analysis, tumours harbouring *KRAS* mutation in either baseline biopsy or post-treatment resection specimen were considered as *KRAS* mutant. Similar approach was used for *NRAS* and *BRAF* mutation.

Further to the results of the ctDNA analysis, ddPCR as described above was performed on the same DNA which was previously extracted for standard PCR-based techniques as well as on DNA which was extracted from different FFPE tissue sections (if available) in order to screen for *KRAS* mutations which were found in the plasma but not previously detected in the tissue.

All methods were carried out in accordance with the Human Tissue Act 2004. The study protocol including molecular analyses on tumour tissues and blood samples was approved by the Committee for Clinical Research at The Royal Marsden NHS Foundation Trust and the relevant Research Ethics Committee. Written informed consent was obtained from all subjects.

### Statistical analysis

The primary endpoint of the EXPERT-C trial was complete response (CR, either pathologic or clinical) in patients with *KRAS* exon 2–3*/BRAF* wild-type tumours and the study was designed to detect a 20% improvement with the addition of cetuximab (odds ratio 3.4, two-sided α of 5% and 80% power). Secondary endpoints included progression-free survival (PFS) and overall survival (OS)^25^.

The main objective of this analysis was to assess the proportion of patients with detectable ctDNA using somatic *KRAS/BRAF* mutations as detection markers. Other objectives included evaluation of the association between ctDNA, clinico-pathological characteristics and outcome in *KRAS* mutant patients and the impact of ctDNA detection in relation to the short- and long-term effects of cetuximab in the entire study population.

Potential biases in the selection of eligible patients for this retrospective study were investigated by comparing the distribution of known prognostic clinical variables and treatment allocation between patients included in the analysis and those excluded due to lack of suitable blood samples. χ^2^ test or Fisher’s exact test were used to investigate potential associations between patient groups and categorical variables whilst t-test or non-parametric equivalent tests were used for continuous variables. Analyses were mostly descriptive. CR was defined using the RECIST v1.1 criteria. PFS was measured from date of randomisation to date of first progression/relapse or death from any cause. OS was measured from date of randomisation to death from any cause. Patients without an event were censored at last date known to be alive. The Kaplan-Meier method was used to summarise the survival estimates while the Cox proportional hazards model was used to compare the survival rates between patient groups with and without adjustment for the effect of covariates. The proportional hazards assumption was tested with the use of Schoenfeld residuals. Interaction tests were conducted to investigate whether a differential treatment effect (i.e., with or without cetuximab) was present for CR rate, PFS and OS within the patient groups analysed.

## Results

**Figure 1:**
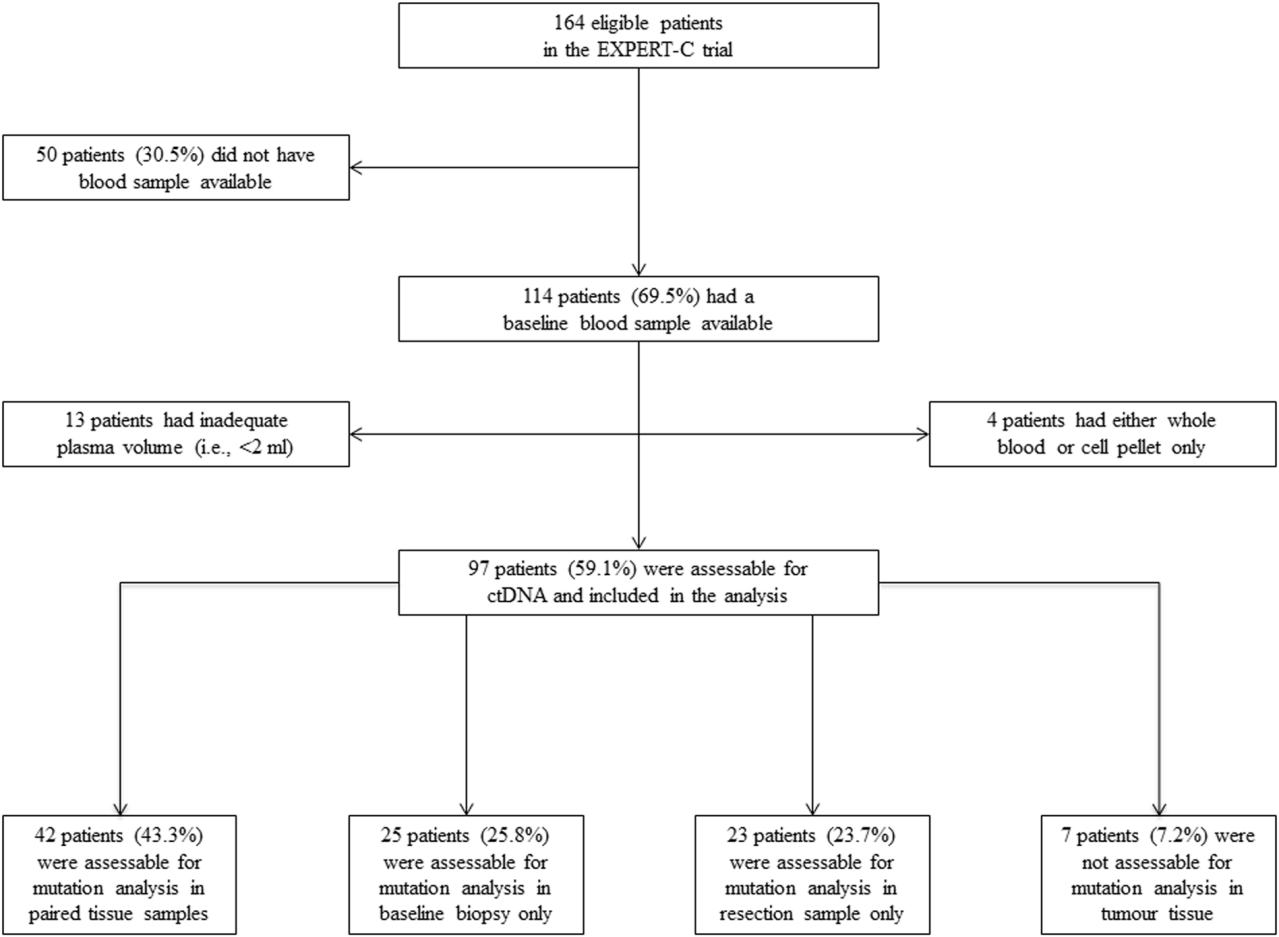
Study flow diagram and tissue availability in the ctDNA assessable population.

Of 164 eligible patients who were randomised in the EXPERT-C trial, 97 (59%) were assessable for the analysis of *KRAS/BRAF* mutations in ctDNA (Fig. 1). There were no statistically significant differences in terms of demographics or baseline patient/tumour characteristics between the assessable and non-assessable groups with the only exception of location of the primary tumour and nodal status as defined by high-resolution MRI (Supplementary Table 1). Outcome was also similar between the two groups with regard to CR rate [OR 0.91 (95% CI: 0.40–2.06), p = 0.81], PFS [HR 1.21 (95% CI: 0.70–2.10), p = 0.50] and OS [HR 1.45 (95% CI: 0.78–2.69), p = 0.24].

Information on the tissue *KRAS/BRAF* status was available for the majority of ctDNA assessable patients (i.e., 90/97, 93%; lack of samples in 4 cases, analysis failed in 3 cases). Overall, *KRAS* mutations were previously detected in 38 patients (42%). Twenty-eight had a mutation in codon G12D, G12V or G13D (31%). Less common *KRAS* mutations were found in 10 additional patients (i.e., G12C = 3, G12S = 3, G12A = 2, A146T = 2) (11%). Paired tumour samples were available in 42 cases and the concordance for *KRAS* status was 83% (25 *KRAS* wild-type and 10 *KRAS* mutant) while in 7 patients *KRAS* mutation was detected in the pre-treatment biopsy but not in the post-treatment resection specimen. Only 3 patients (3%) were found to have *BRAF* mutant tumours (in 2 cases the mutation was detected in paired samples while in 1 case in the pre-treatment biopsy only).

**Figure 2:**
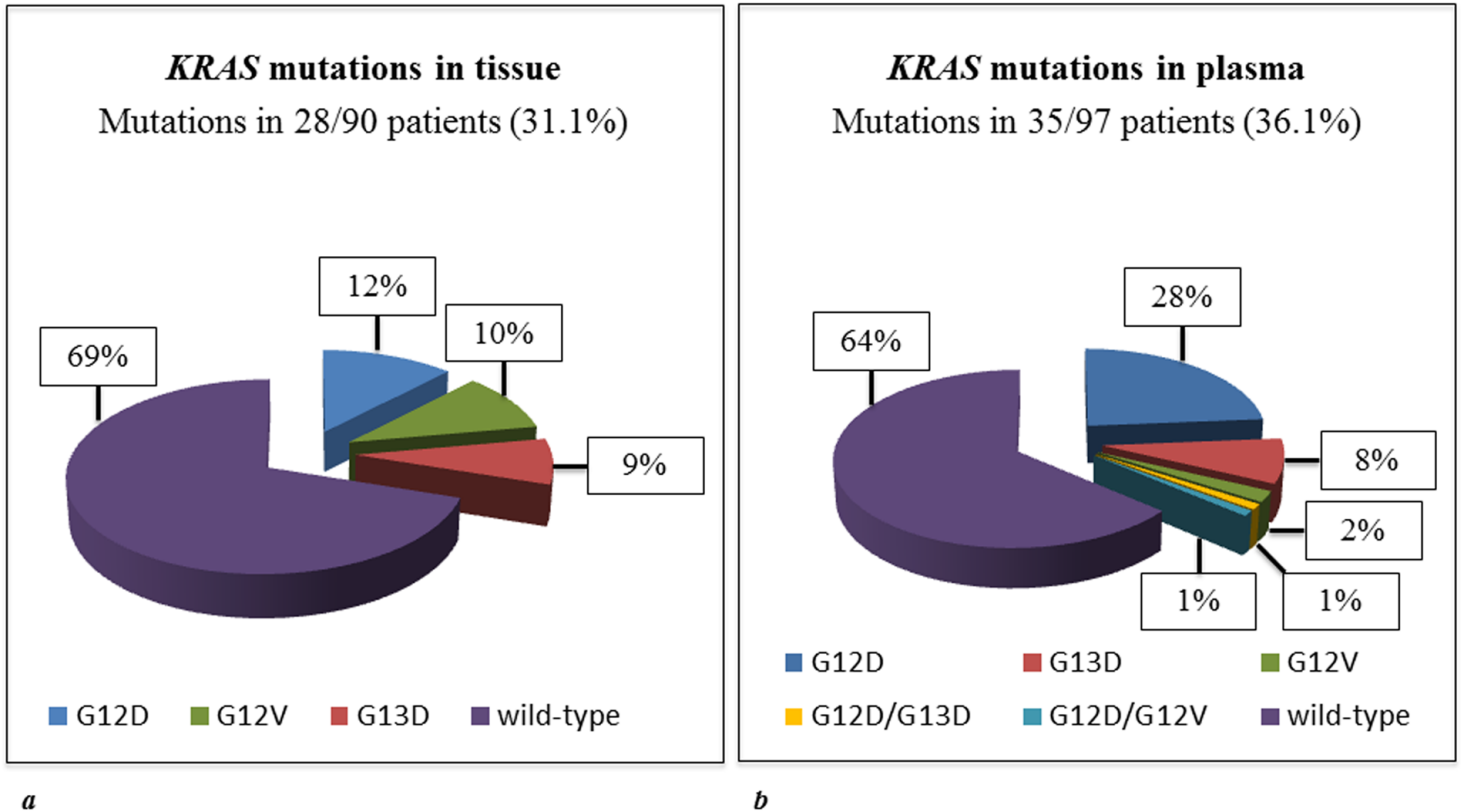
Frequency of the most frequently mutated *KRAS* hotspot mutation (*i.e*., G12D, G12V and G13D) in tissue (**a**) and plasma (**b**) of patients who were assessable for the analysis of circulating tumour DNA (tissue *KRAS* status was unknown for 7/97 patients).

Mutations in codon G12D, G12V or G13D were found in the ctDNA of 35/97 assessable patients (36%) and 34/90 (38%) patients with known tissue *KRAS* status (Fig. 2). In 2 patients two mutations were found (i.e., G12D/G13D and G12D/G12V). The median mutant allele frequency in ctDNA was 0.41% for G12D (range 0.02–5.88), 0.46% for G12V (range 0.06–2.35) and 0.22% for G13D (range 0.04–4.07). While the frequency of G13D mutation appeared similar between tissue and blood samples (i.e., 9% *vs*. 9%, p = 0.92), G12D mutations were detected more frequently (i.e., 26% *vs*. 12%, p = 0.02) and G12V less frequently (i.e., 3% *vs*. 10%, p = 0.06) in ctDNA compared with tumour tissue. Mutation in codon V600E of the *BRAF* gene was found in the ctDNA of 2 patients (2%).

Table 1 shows the comparison of *KRAS* status between tissue and plasma samples of individual patients (analysis restricted to codon G12D, G12V and G13D). A mutation in any of these 3 codons was found in the ctDNA of 12/28 patients (43%) with *KRAS* mutant tumours. In 3 cases the mutation involved a different codon compared with that which was previously detected in the tissue (Supplementary Table 2). In the group of 62 patients with *KRAS* wild-type tumours, plasma mutations in G12D, G12V or G13D were identified in 22 cases (35%). Of note, in 11 of these (50%) the absence of tissue *KRAS* mutation was previously confirmed in both baseline biopsy and resection samples (Supplementary Table 3). The concordance rate between tissue *KRAS* mutation analysis by standard PCR-based techniques and ctDNA analysis by ddPCR was 58% (52/90) and 54% (49/90) for the overall mutational status and specific mutations, respectively. Among the 10 patients with less common *KRAS* mutation (G12C, G12S, G12A or A146T), the ctDNA detection rate was 50% (5/10). In 2 patients, a new mutation in G12D was found instead of, or in addition to, the mutation which was previously detected in the tumour tissue (Supplementary Table 4). When data on less common mutations were also considered, the concordance rate between tissue *KRAS* mutation analysis by standard PCR-based techniques and ctDNA analysis by ddPCR was 56% (50/90) and 46/90 (51%), for the overall mutational status and specific mutations, respectively (Supplementary Table 5). *BRAF* mutation was detected in the ctDNA of 1 out of 3 patients with *BRAF* mutant tumours and in 1 additional patient with a *KRAS* mutant/*BRAF* wild-type tumour.

**Table 1:** Comparison of *KRAS* status (analysis restricted to codon G12D, G12V and G13D) between paired tissue (analysed by standard PCR-based techniques) and plasma samples (analysed by ddPCR).

*Blood*	*KRAS* WT	*KRAS* MUT	Total
***Tissue***			
*KRAS* WT	40 (64.5%)	22 (35.5%)^*^	62 (63.9%)
*KRAS* MUT	16 (57.1%)	12 (42.9%)^**^	28 (28.9%)
*KRAS* UNK	6 (85.7%)	1 (14.3%)	7 (7.2%)
Total	62 (63.9%)	35 (36.1%)	97 (100%)

^*^In 11 cases the absence of tissue *KRAS* mutation was previously confirmed in both baseline biopsy and resection samples.

^**^In 3 cases the *KRAS* mutation which was found in ctDNA involved a different codon compared with the *KRAS* mutation which was previously detected in the tissue.

Abbreviations: WT: wild-type; MUT: mutant; UNK: unknown.

**Figure 3:**
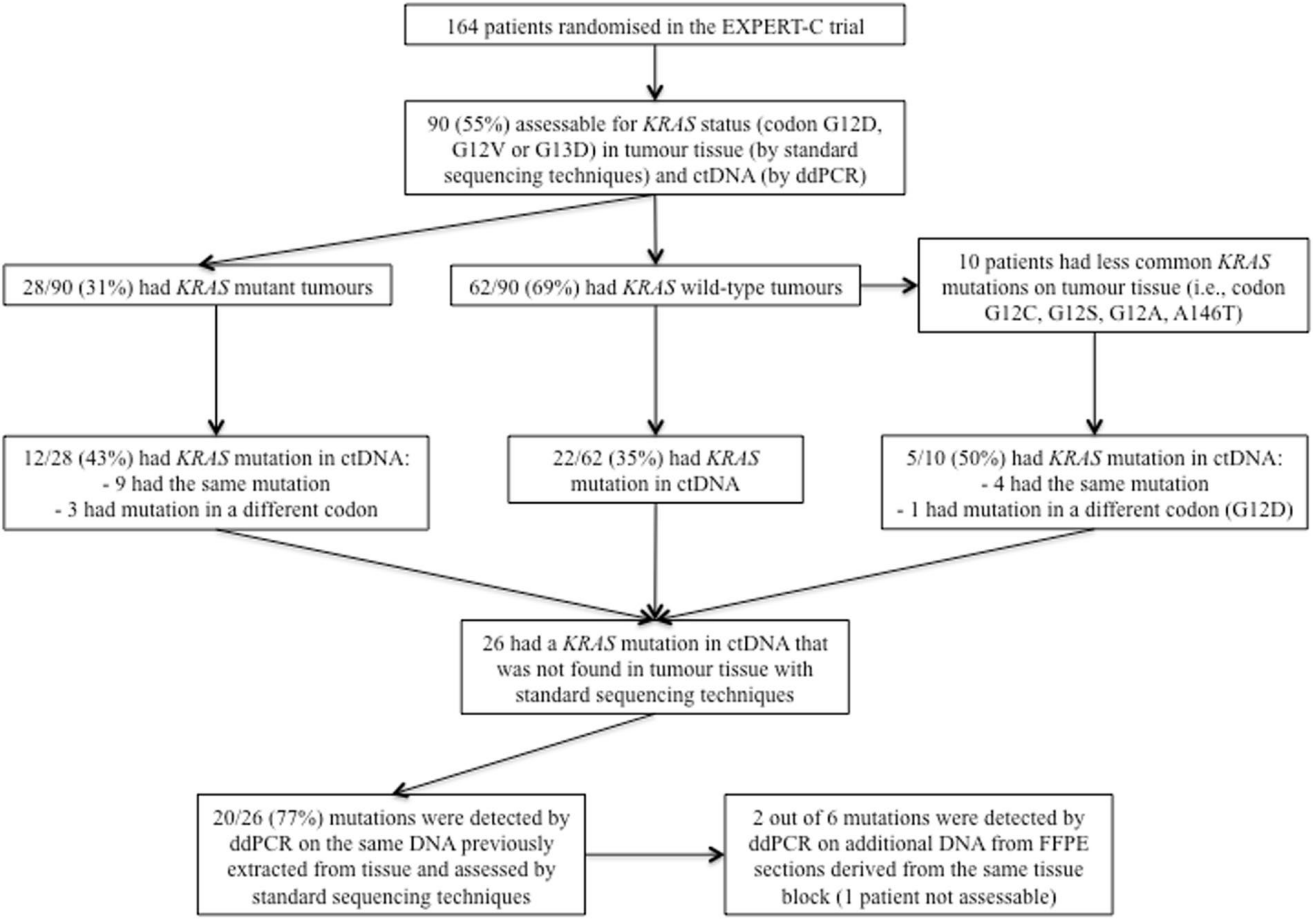
Results of *KRAS* mutational analysis by standard PCR-based techniques and ddPCR in tissue and plasma.

Overall, in 26 cases ctDNA analysis revealed a *KRAS* mutation that was not previously found in tumour tissue (i.e., 22 patients previously classified as having *KRAS* wild-type tumours and 4 patients with *KRAS* mutant tumours who were found to have a different *KRAS* mutation in ctDNA). In some of these patients, the discrepancy between the results of tissue and plasma mutational analysis could be partly explained by a number of factors such as low frequency of the mutant allele in plasma, limited tissue availability (i.e., only one sample available, either biopsy or resection) or low tumour infiltration in tissue specimens (Supplementary Tables 6 and 7). Nevertheless, when ddPCR was used to analyse the same DNA that was previously extracted from tissue and assessed by standard PCR-based techniques, 20 of these 26 apparently newly detected plasma mutations (77%) were also detectable in the tissue. For 5 of the 6 patients with still discordant results, additional DNA from FFPE sections derived from the same tissue block was available for ddPCR testing. Two of these patients were confirmed to have tumours harbouring a *KRAS* mutation for an overall concordance between tissue and plasma of 84% (22/26) (Fig. 3). In the remaining 4 patients in whom no *KRAS* mutation was detectable in tissue, the median mutant allele frequency in plasma was 0.64 (range 0.09–1.10).

When the results of *KRAS/BRAF* tissue analysis by ddPCR were taken into account, 59 patients were found to have mutant tumours for either *KRAS* or *BRAF* (mutations being detected in 38 cases by standard PCR-based techniques while in 21 cases by ddPCR). In this population the ctDNA detection rate was 66% (39/59). In Table 2 demographics and baseline characteristics of *KRAS/BRAF* mutant/ctDNA negative patients (n = 20) are compared with those of *KRAS/BRAF* mutant/ctDNA positive patients (n = 39). No differences were observed between these groups with the only exception of MRI T stage at baseline (p = 0.01). In particular, 25% of patients in the ctDNA negative group had T3d/T4 tumours compared with 47% of patients in the ctDNA positive group. EMVI was observed in 74% of ctDNA positive versus 55% of ctDNA negative patients while tumour location above levator muscles was observed in 44% and 20% in ctDNA positive and negative patients respectively but none of these differences were statistically significant. The analysis of outcome measures showed no difference between ctDNA positive and ctDNA negative patients in terms of CR rate [i.e., 15.4% *vs*. 10%, OR 1.63 (95% CI: 0.30–8.96), p = 0.57], PFS [HR 0.70 (95% CI: 0.29–1.70), p = 0.43] and OS [HR 0.78 (95% CI: 0.31–1.96), p = 0.60]. Adjustment for known prognostic clinical variables did not change the results.

**Table 2:** Comparison of demographics and baseline characteristics between *KRAS* mutant/ctDNA negative patients (n = 20) and *KRAS* mutant/ctDNA positive patients (n = 39).

	ctDNA neg (n = 20)		ctDNA pos (n = 39)		*p* value
	N	%	N	%	
**Gender**					
Male	13	65	23	59	0.65
Female	7	35	16	41	
**Age**					
Median (IQR)	66.7 (55.5–74.4)		65.1 (59.2–68.6)		0.38
**WHO PS**					
0	10	50	18	46	0.78
1–2	10	50	21	54	
**MRI T**					
T3a	1	5	0	0	0.01
T3b	1	5	6	15	
T3c	13	65	15	38	
T3d	0	0	10	26	
T4a	4	20	3	8	
T4b	1	5	5	13	
**MRI EMVI+**					
No	9	45	10	26	0.13
Yes	11	55	29	74	
**MRI CRM+**					
No	11	55	20	51	0.79
Yes	9	45	19	49	
**Tumour height**					
At/below lev	16	80	22	56	0.09
Above lev	4	20	17	44	
**MRI N status**					
N0	6	30	15	38	0.28
N1	8	40	8	21	
N2	6	30	16	41	
**Treatment arm**					
CAPOX	12	60	21	54	0.65
CAPOX-C	8	40	18	46	

Abbreviations: WHO: World Health Organisation; PS: performance status; MRI: magnetic resonance imaging; EMVI: extramural venous invasion; CRM: circumferential resection margin; lev: levator muscles.

Overall availability of biological samples for mutational analysis in the EXPERT-C trial (n = 164) was as follows: 90 patients (55%) were assessable in both tissue and plasma, 59 (36%) in tumour tissue only and 7 (4%) in plasma only. Eight patients (5%) were not assessable in either tissue or plasma. In an exploratory analysis, data on *KRAS* status in ctDNA were combined with previously obtained data on *KRAS*/*NRAS* status in tumour tissue and patient outcome assessed by presence/absence of these mutations. Demographics and baseline characteristics were well balanced between patients with mutant tumours (i.e., *KRAS*/*NRAS* mutation in tissue and/or *KRAS* mutation in blood, n = 92) and those with wild-type tumours (i.e., neither *KRAS*/*NRAS* mutation in tissue nor *KRAS* mutation in blood, n = 30). No differences between these groups were observed with respect to CR rate (i.e., 15.2% *vs*. 10.0%, OR 1.62 (95% CI: 0.43–6.05), p = 0.48), PFS [HR 0.90 (95% CI: 0.47–1.75), p = 0.77] and OS [HR 1.01 (95% CI: 0.47–2.14), p = 0.98]. Also, no interaction was observed between mutation status and outcome of cetuximab treatment for CR (p = 0.52), PFS (p = 0.59) and OS (p = 0.98). Among the 90 patients with available data on *KRAS/NRAS* status on tissue and *KRAS* status on plasma, 8 (9%) had at least 2 *RAS* mutations (i.e., 2 mutations in 8 patients, 3 mutations in 1 patient). Of these, 5 experienced tumour recurrence following curative treatment, 2 were alive and disease-free at 5 years and 1 withdrew the study due to toxicity.

## Discussion

While the potential application of ctDNA analysis in early stage colon cancer and advanced colorectal cancer has been increasingly reported, the clinical utility of liquid biopsy in the setting of non-metastatic rectal cancer has been so far the subject of limited investigation. To our knowledge only 4 small studies addressing this topic have been published.

By using a real time PCR technique Zitt *et al*. analysed the levels of circulating cell-free DNA in 26 patients who were treated with pre-operative, fluorouracil-based, chemoradiotherapy for cT3–4 mid-low tumours^21^. A reduction of DNA levels was observed after completion of chemoradiotherapy in all patients but neither baseline nor post-treatment DNA concentrations were predictive of pathological downstaging. A significant difference between responders (i.e., ypT0–2) and non-responders (i.e., ypT3–4) was found only after surgery (i.e., further decrease of DNA in responders but increase of DNA in non-responders, p = 0.006). In a larger study including 67 patients with cT3-4 and/or N+ tumours, Agostini *et al*. showed that the circulating cell-free DNA integrity index (i.e., a ratio between long and short DNA fragments) was statistically significantly different between responding and non-responding patients (as defined by the degree of tumour regression according to the Mandard score) only after completion of fluoropyrimidine-based chemoradiotherapy (p = 0.0009) but not at baseline^22^. The DNA integrity index at this time point was also found to be the only independent predictive factor of response to neoadjuvant treatment in multivariate analysis. The potential of the DNA integrity index in this setting was confirmed by Sun *et al*. who showed an association between this parameter (both at baseline and after neoadjuvant treatment) and pathological tumour regression grading according to the Dworak’s score in 34 patients who received an oxaliplatin-based chemoradiotherapy for cT3-4 and/or N+ rectal tumours^23^. Interestingly, while the rate of *KRAS* codon 12 mutation decreased with chemoradiotherapy in all patients with no difference between responders and non-responders, a higher rate of MGMT methylation at baseline was predictive of pathological response. Finally, in a recent report of 4 LARC patients whose ctDNA was tracked in serial blood samples by using two patient-specific chromosomal rearrangements, Carpinetti *et al*. showed an overall lack of correlation between normalisation of ctDNA and amount of residual disease in the surgical specimens after neoadjuvant chemoradiotherapy^24^. However, changes of ctDNA levels after surgery appeared to predict tumour recurrence.

The aims of our study were to confirm that ctDNA analysis is feasible in a population of non-metastatic rectal cancer patients, to assess *KRAS/BRAF* mutations as markers for ctDNA detection, and to explore potential clinical implications of detectable *KRAS* mutations in ctDNA. For this purpose, we analysed a relatively large and homogeneous series of patients with MRI-defined, high-risk, LARC who were treated with an investigational strategy plus or minus cetuximab within a randomised phase II trial^25^. The results support the potential of ctDNA as biomarker for LARC based on a number of interesting findings. Also they highlight possible challenges for future prospective ctDNA-based studies in this setting.

By using a pragmatic approach and restricting our analysis to the three most common *KRAS* mutations in exon 2 in the overall study population and to less common, patient-specific *KRAS* mutations in selected cases, we initially found that ctDNA was detectable in approximately 40% of patients. Interestingly, the rate of *KRAS* mutation in plasma did not appear much different between patients with *KRAS* wild-type and those with *KRAS* mutant tumours. Also, in some patients from the latter group, the analysis of ctDNA showed the presence of additional *KRAS* mutations that were not detected on tissue. These observations support the higher sensitivity of ddPCR compared with the standard PCR-based techniques that were originally used for tumour mutational analysis in our series^27^. Indeed, when tissue-derived DNA of patients with discordant results was assessed by ddPCR, high concordance for *KRAS* status (i.e., 85%) was observed between tissue and plasma. On the other hand, the lack of complete concordance between tissue and plasma (i.e., 15% of patients had *KRAS* mutation in plasma only) suggests the ability ctDNA analysis to provide a more comprehensive characterisation of tumour molecular profiling and minimise the impact of tumour heterogeneity/random tissue sampling on routine mutational testing procedures. In support of this contention, in 2 out of 22 patients with concordant results, the *KRAS* mutation was found only after the analysis of DNA which was extracted from different FFPE tissue sections.

Generally, one of the main concerns regarding the use of highly sensitive techniques is the clinical significance of sub-clonal somatic mutations that occur at a low frequency and therefore would be below the detection threshold of common diagnostic platforms which are used in routine practice. Studies in metastatic colorectal cancer, however, suggest that increasing the detection sensitivity for tumour *KRAS* mutations may actually refine patient selection for anti-EGFR monoclonal antibodies by identifying a higher proportion of patients who fail to respond to these agents^28^. Furthermore, detection of low-frequency *KRAS* mutant alleles in blood of patients treated with cetuximab or panitumumb may represent an early sign of expansion/emergence of tumour resistant clones, predict forthcoming clinical progression and warrant consideration of alternative treatment strategies to overcome resistance^7,17^. In this study, we could not demonstrate that refining the *RAS* wild-type population by taking into account the results of the ctDNA analysis had an impact on the overall effect of cetuximab. In line with our previous reports of this trial where patients were classified according to either *KRAS* exon 2–3*/BRAF* or extended *RAS* tissue mutational status^25,29,30^, adding cetuximab to chemotherapy and chemoradiotherapy did not significantly improve the outcome of wild-type tumours. It should be noted, however, that in the EXPERT-C trial the use of cetuximab was investigational and there is still no evidence that EGFR is a valuable therapeutic target for LARC. Furthermore, some of the limitations of this study (i.e., retrospective analysis and relatively small sample size) and the use of a multimodality treatment approach including radiotherapy may have introduced significant biases and limited the ability to assess the role of either *KRAS* or *RAS* mutations as negative predictive biomarkers for cetuximab in this setting.

Despite the high sensitivity of ddPCR, more than one third of patients with *KRAS/BRAF* mutant tumours in our study were not found to harbour the corresponding mutation in plasma. One could argue that this may be partly explained by the decision to consider data from the analysis of resection specimens as representative of the tumour mutational status even in the absence of an assessable biopsy sample at baseline. However, the degree of concordance between paired specimens in our series was high and there was no case where the emergence of *KRAS* mutant clones following neoadjuvant treatment could be suspected. Furthermore, it is important to note that our data are in line with those previously reported in series of non-metastatic patients with colorectal cancer or other tumour types^11,31,32^. Overall, this indicates that alternative strategies could be necessary to exploit the clinical potential of ctDNA analysis in larger populations. Although undetectable plasma *KRAS/BRAF* mutations could just reflect the absence of micrometastatic disease, ctDNA assays designed to detect multiple tumour-specific somatic aberrations may prove to be more sensitive and useful than those detecting single gene mutations (especially if present at a low allelic frequency). In this regard, it is interesting to note that, in our study, a difference between tissue and plasma was noted in terms of relative frequency of specific *KRAS* mutations. While this could be a random effect, it may also suggest that, at least in the setting of non-metastatic disease/limited tumour burden, tumours with specific *KRAS* mutations (i.e., G12V) are less likely to release DNA in blood compared to those harbouring different mutations (i.e., G12D).

Although studies suggest that ctDNA analysis could be used as a minimally invasive, dynamic tool to predict prognosis and response to adjuvant treatment in localised colorectal cancer^10–12^, the results of our analysis do not appear to support this contention. In fact, even though an association between baseline poor-prognosis features and detectable ctDNA such as advanced T stage was found, there was no difference in outcome between patients with or without detectable ctDNA. It is possible, however, that this apparent discrepancy may be due to the timing of blood sampling (i.e., pre-operative in our study vs. post-operatively in other series) and the biological difference between detectable ctDNA at each of these time points. Furthermore, the treatment strategy used in the EXPERT-C trial with upfront administration of systemic chemotherapy might have limited the negative prognostic impact of ctDNA, this hypothesis being supported by the relatively low incidence of distant metastases observed in this trial and in other similar studies^30,33^.

Our study is the largest analysis of ctDNA in non-metastatic rectal cancer. It suffers, however, from a number of limitations such as the retrospective approach, the limited number of assessable patients and the use of detection techniques with different sensitivity in tumour tissue and plasma. Furthermore, the availability of only one blood sample per patient at baseline precluded the analysis of changes of ctDNA during/after neoadjuvant treatment which are likely to be more informative in terms of assessment of treatment response and consideration of adaptive treatment strategies. Nevertheless, the data here reported confirm that *KRAS/BRAF* mutations in ctDNA can be detected by ddPCR with high sensitivity and provide further support for the initiation of prospective trials investigating the potential clinical applications of ctDNA analysis in the setting of LARC.

## Electronic supplementary material

Supplementary Tables
