# Supplementary material for: KRAS and BRAF mutations in circulating tumour DNA from locally advanced rectal cancer
Source: Sci Rep. 2018 Jan 23;8:1445. doi: 10.1038/s41598-018-19212-5 (PMC5780472; doi:10.1038/s41598-018-19212-5)
Supplement: Supplementary file 1 — Supplementary Tables [file 41598_2018_19212_MOESM1_ESM.doc]

**Supplementary files**

***KRAS* and *BRAF* mutations in circulating tumour DNA from locally advanced rectal cancer**

Francesco Sclafani^1^, Ian Chau^1^, David Cunningham^1^, Jens C Hahne^2^, George Vlachogiannis^2^, Zakaria Eltahir^1^, Andrea Lampis^2^, Chiara Braconi^1,2^, Eleftheria Kalaitzaki^1^, David Gonzalez de Castro^1^, Andrew Wotherspoon^1^, Jaume Capdevila^3^, Bengt Glimelius^4^, Noelia Tarazona^5^, Ruwaida Begum^1^, Hazel Lote^1,2^, Sanna Hulkki Wilson^1^, Giulia Mentrasti^2^, Gina Brown^1^, Diana Tait^1^, Jacqui Oates^1^, Nicola Valeri^1,2^

Authors’ affiliation

^1^ The Royal Marsden NHS Foundation Trust, London and Surrey, United Kingdom.

^2^ The Institute of Cancer Research, London and Surrey, United Kingdom.

^3^ Vall d’Hebron University Hospital, Universitat Autònoma de Barcelona, Barcelona, Spain

^4^ University of Uppsala, Uppsala, Sweden

^5^ Biomedical Research Institute INCLIVA, University of Valencia, Spain

**Supplementary Table 1.** Comparison between patients who were assessable for the analysis of circulating tumour DNA and those who were not with regard to demographics, baseline characteristics and outcome

|  | **Not assessable (n=67)** | | **Assessable (n=97)** | | *p* value |
| --- | --- | --- | --- | --- | --- |
|  | **N** | **%** | **N** | **%** |  |
| **Gender** | | | | | |
| Male | 43 | 64 | 58 | 60 | 0.57 |
| Female | 24 | 36 | 39 | 40 |  |
| **Age** | | | | | |
| Median (sd) | 60.4 (10.8) | | 62.1 (10.8) | | 0.32 |
| **WHO PS** | | | | | |
| 0 | 29 | 43 | 49 | 51 | 0.36 |
| 1-2 | 38 | 57 | 48 | 49 |  |
| **MRI T3c/d** | | | | | |
| No | 27 | 40 | 34 | 35 | 0.49 |
| Yes | 40 | 60 | 63 | 65 |  |
| **MRI T4** | | | | | |
| No | 50 | 75 | 74 | 76 | 0.81 |
| Yes | 17 | 25 | 23 | 24 |  |
| **MRI EMVI+** | | | | | |
| No | 18 | 27 | 28 | 29 | 0.78 |
| Yes | 49 | 73 | 69 | 71 |  |
| **MRI CRM+** | | | | | |
| No | 24 | 36 | 47 | 48 | 0.11 |
| Yes | 43 | 64 | 50 | 52 |  |
| **Tumour height** | | | | | |
| At/below lev | 57 | 85 | 64 | 67 | 0.01 |
| Above lev | 10 | 15 | 32 | 33 |  |
| **MRI N status** | | | | | |
| N0 | 17 | 25 | 30 | 31 | 0.04 |
| N1 | 30 | 45 | 25 | 26 |  |
| N2 | 20 | 30 | 42 | 43 |  |
| **Treatment arm** | | | | | |
| CAPOX | 30 | 45 | 51 | 53 | 0.33 |
| CAPOX-C | 37 | 55 | 46 | 47 |  |
| **Outcome measures** | | | | | |
| pCR | 12 | 17.9 | 16 | 16.5 | 0.81 |
| PFS | HR 1.21 (95% CI: 0.70 - 2.10)^*^ | | | | 0.50 |
| OS | HR 1.45 (95% CI: 0.78 - 2.69)^*^ | | | | 0.24 |

^*^ The HR refers to assessable patients *vs.* those not assessable.

***Abbreviations:*** *sd: standard deviation; WHO: World Health Organisation; PS: performance status; MRI: magnetic resonance imaging; EMVI: extramural venous invasion; CRM: circumferential resection margin; lev: levator muscles; pCR: pathological complete response; PFS: progression-free survival; OS: overall survival; HR: hazard ratio; CI: confidence interval.*

**Supplementary Table 2.** Analysis of G12D, G12V and G13D *KRAS* mutation in ctDNA of patients with *KRAS* mutant tumours

| **Patient ID** | ***KRAS* status on DNA from pre-treatment biopsy sample** | ***KRAS* status on**  **pre-treatment ctDNA** | ***KRAS* status on DNA from post-treatment resection sample** |
| --- | --- | --- | --- |
| 1001 | UNK | MUT (G13D) | MUT (G13D) |
| 1005 | MUT (G12V) | WT | WT |
| 1006 | MUT (G12D) | MUT (G12D) | UNK |
| 1007 | MUT (G12D) | WT | MUT (G12D) |
| 1008 | MUT (G12D) | WT | UNK |
| 1018 | MUT (G12D) | MUT (G12D) | WT |
| 1020 | MUT (G12V) | WT | MUT (G12V) |
| 1024 | MUT (G12V) | MUT (G12D) | UNK |
| 12002 | MUT (G12D) | WT | UNK |
| 31010 | UNK | WT | MUT (G12D) |
| 31015 | MUT (G12V) | MUT (G13D) | MUT (G12V) |
| 31019 | MUT (G12V) | WT | UNK |
| 31020 | UNK | MUT (G12D) | MUT (G12D) |
| 31023 | MUT (G12V) | WT | UNK |
| 31024 | UNK | MUT (G13D) | MUT (G13D) |
| 32007 | MUT (G12D) | WT | UNK |
| 51001 | MUT (G12V) | WT | WT |
| 51005 | MUT (G13D) | WT | UNK |
| 132003 | MUT (G12V) | WT | UNK |
| 141002 | MUT (G12D) | WT | UNK |
| 141006 | MUT (G13D) | MUT (G12D/G13D) | WT |
| 142002 | MUT (G13D) | MUT (G13D) | UNK |
| 142004 | MUT (G13D) | MUT (G13D) | MUT (G13D) |
| 151001 | MUT (G12V) | MUT (G13D) | UNK |
| 151002 | MUT (G12D) | WT | UNK |
| 152001 | MUT (G13D) | MUT (G13D) | UNK |
| 171004 | MUT (G12D) | WT | MUT (G12D) |
| 172001 | MUT (G13D) | WT | UNK |

***Abbreviations:*** *ID: identifier; WT: wild-type; MUT: mutant; UNK: unknown.*

**Supplementary Table 3.** Analysis of G12D, G12V and G13D *KRAS* mutation in ctDNA of patients with *KRAS* wild-type tumours

| **Patient ID** | ***KRAS* status on DNA from pre-treatment biopsy sample** | ***KRAS* status on**  **pre-treatment ctDNA** | ***KRAS* status on DNA from post-treatment resection sample** |
| --- | --- | --- | --- |
| 1002 | WT | MUT (G12D) | WT |
| 1004 | WT | MUT (G12V) | WT |
| 1009 | WT | WT | WT |
| 1010 | WT | MUT (G12D) | UNK |
| 1011 | WT | WT | UNK |
| 1013 | WT | WT | WT |
| 1014 | WT | WT | WT |
| 1016 | WT | WT | WT |
| 1017 | UNK | MUT (G12D) | WT |
| 1019 | WT | MUT (G13D) | UNK |
| 1021 | WT | WT | WT |
| 1022 | WT | WT | WT |
| 1023 | WT | MUT (G12D) | WT |
| 1025 | WT | WT | WT |
| 11027 | WT | MUT (G12D/G12V) | WT |
| 11030 | WT | MUT (G12D) | WT |
| 12001 | WT | WT | UNK |
| 12003 | WT | MUT (G12D) | UNK |
| 12004 | UNK | WT | WT |
| 31005 | WT | MUT (G12V) | WT |
| 31006 | WT | WT | WT |
| 31009 | UNK | WT | WT |
| 31011 | WT | WT | WT |
| 31012 | WT | WT | WT |
| 31013 | UNK | WT | WT |
| 31014 | UNK | WT | WT |
| 31016 | WT | WT | UNK |
| 31017 | UNK | MUT (G12D) | WT |
| 31021 | WT | WT | WT |
| 31022 | UNK | WT | WT |
| 32001 | UNK | MUT (G12D) | WT |
| 32002 | UNK | WT | WT |
| 32003 | UNK | WT | WT |
| 32004 | UNK | WT | WT |
| 32006 | UNK | WT | WT |
| 32008 | WT | WT | WT |
| 51002 | WT | WT | WT |
| 51003 | WT | WT | WT |
| 71002 | WT | WT | WT |
| 111001 | WT | MUT (G12D) | WT |
| 131001 | WT | MUT (G12D) | WT |
| 131002 | WT | MUT (G12D) | WT |
| 131004 | WT | MUT (G12D) | WT |
| 131006 | WT | WT | UNK |
| 131009 | UNK | MUT (G12D) | WT |
| 131010 | WT | WT | WT |
| 132001 | WT | MUT (G12D) | WT |
| 132002 | WT | WT | UNK |
| 132004 | UNK | MUT (G12D) | WT |
| 132005 | WT | WT | WT |
| 141001 | UNK | MUT (G12D) | WT |
| 141003 | UNK | WT | WT |
| 141005 | WT | WT | UNK |
| 141008 | UNK | WT | WT |
| 141009 | WT | WT | WT |
| 142001 | WT | WT | WT |
| 142003 | WT | WT | UNK |
| 142005 | UNK | MUT (G12D) | WT |
| 152002 | WT | WT | WT |
| 171001 | WT | WT | WT |
| 171002 | WT | MUT (G12D) | UNK |
| 171003 | WT | WT | WT |

***Abbreviations:*** *ID: identifier; WT: wild-type; MUT: mutant; UNK: unknown.*

**Supplementary Table 4.** Comparison of *KRAS* status between tissue and plasma of patients who were known to have tissue *KRAS* mutations in codons other than G12D, G12V and G13D

| **Patient ID** | ***KRAS* status on DNA from pre-treatment biopsy sample** | ***KRAS* status on**  **pre-treatment ctDNA** | ***KRAS* status on DNA from post-treatment resection sample** |
| --- | --- | --- | --- |
| 1022 | MUT (G12C) | MUT (G12C) | MUT (G12C) |
| 12004 | UNK | MUT (G12A) | MUT (G12A) |
| 31012 | MUT (A146T) | WT | WT |
| 51002 | MUT (A146T) | WT | WT |
| 131010 | MUT (G12C) | WT | WT |
| 132001 | MUT (G12S) | MUT (G12S/G12D) | MUT (G12S) |
| 132005 | MUT (G12S) | WT | MUT (G12S) |
| 142001 | MUT (G12C) | MUT (G12C) | MUT (G12C) |
| 142003 | MUT (G12S) | MUT (G12S) | MUT (G12S) |
| 171002 | MUT (G12A) | MUT (G12D) | UNK |

***Abbreviations:*** *ID: identifier; WT: wild-type; MUT: mutant; UNK: unknown.*

**Supplementary Table 5.** Comparison of *KRAS* status (analysis restricted to codon G12D, G12V and G13D for all samples and any patient-specific, additional *KRAS* mutation which was previously detected in the tumour tissue) between paired tissue (analysed by standard PCR-based techniques) and plasma samples (analysed by ddPCR)

|  | ***Blood*** | ***KRAS* WT** | ***KRAS* MUT** | **Total** |
| --- | --- | --- | --- | --- |
| ***Tissue*** |  |
| ***KRAS* WT** | | 32 (61.5%) | 20 (38.5%)^*^ | 52 (53.6%) |
| ***KRAS* MUT** | | 20 (52.6%) | 18 (47.4%)^**^ | 38 (39.2%) |
| ***KRAS* UNK** | | 6 (85.7%) | 1 (14.3%) | 7 (7.2%) |
| **Total** | | 58 (59.8%) | 39 (40.2%) | 97 (100%) |

^*^ In 11 cases the absence of tissue *KRAS* mutation was previously confirmed in both baseline biopsy and resection samples

^**^ In 5 cases, a new mutation was found instead of (n=4), or in addition to (n=1), the mutation which was previously detected in the tumour tissue

***Abbreviations:*** *WT: wild-type; MUT: mutant; UNK: unknown.*

**Supplementary Table 6.** Mutant allele frequency of *KRAS* mutation in ctDNA and tumour infiltration in tissue specimens of patients with *KRAS* mutations in plasma that were not previously detected in tissue (n=26)

| **Patient ID** | **Tumour infiltration**  **in biopsy sample (%)^*^** | **Tumour infiltration in resection sample (%)^*^** | **Mutant allele frequency**  **in plasma (%)** |
| --- | --- | --- | --- |
| 131002 | 70 | 30-50 | 0.03 |
| 1019 | 30-50 | - | 0.04 |
| 141001 | - | <30 | 0.04 |
| 131001 | 30-50 | <30 | 0.05 |
| 1017 | - | 70 | 0.05 |
| 131009 | - | <30 | 0.08 |
| 111001 | 30-50 | <30 | 0.09 |
| 151001 | 30-50 | - | 0.09 |
| 142005 | - | <30 | 0.14 |
| 12003 | 100 | - | 0.26 |
| 1024 | 30-50 | - | 0.28 |
| 132004 | - | <30 | 0.30 |
| 32001 | - | 30-50 | 0.37 |
| 131004 | 30-50 | <30 | 0.41 |
| 132001 | 70 | 30-50 | 0.44 |
| 31005 | 30-50 | 30-50 | 0.46 |
| 171002 | 70 | - | 0.63 |
| 11030 | 70 | <30 | 0.83 |
| 31015 | 30-50 | 30-50 | 1.10 |
| 1002 | <30 | <30 | 1.33 |
| 141006 | - | 30-50 | 1.56 |
| 1010 | 30-50 | - | 2.13 |
| 31017 | - | 30-50 | 2.17 |
| 1004 | 70 | - | 2.35 |
| 1023 | 70 | 70 | 2.44 |
| 11027 | 70 | 30-50 | 5.88 |

^*^ Tumour infiltration in non-microdissected samples

***Abbreviations:*** *ID: identifier.*

**Supplementary Table 7.** Mutant allele frequency of *KRAS* mutation in ctDNA and tumour infiltration in tissue specimens of patients with *KRAS* mutations in plasma that were previously detected in tissue (n=12)

| **Patient ID** | **Tumour infiltration**  **in biopsy sample (%)^*^** | **Tumour infiltration in resection sample (%)^*^** | **Mutant allele frequency**  **in plasma (%)** |
| --- | --- | --- | --- |
| 1006 | 70 | - | 0.02 |
| 151001 | 30-50 | - | 0.09 |
| 1001 | - | 30-50 | 0.13 |
| 142002 | 70 | - | 0.17 |
| 141006 | - | 30-50 | 0.22 |
| 1024 | 30-50 | - | 0.28 |
| 142004 | 70 | 30-50 | 0.90 |
| 152001 | 30-30 | - | 0.97 |
| 31015 | 30-50 | 30-50 | 1.10 |
| 31020 | - | <30 | 1.18 |
| 1018 | na | 50-70 | 1.45 |
| 31024 | **-** | 30-50 | 4.07 |

^*^ Tumour infiltration in non-microdissected samples

***Abbreviations:*** *ID: identifier, na: not available.*
